# Supplementary material for: Floral pathway integrator gene expression mediates gradual transmission of environmental and endogenous cues to flowering time
Source: PeerJ. 2017 Apr 19;5:e3197. doi: 10.7717/peerj.3197 (PMC5399868; doi:10.7717/peerj.3197)
Supplement: Supplemental Information 1 [file peerj-05-3197-s001.docx]

**Floral Pathway Integrator Gene Expression mediates Gradual Transmission of Environmental and Endogenous Cues to Flowering Time**

Aalt D.J. van Dijk^1,2,3*^ and Jaap Molenaar^1^

^1^ Biometris, Wageningen UR, Wageningen, The Netherlands

^2^ Bioscience, Wageningen UR, Wageningen, The Netherlands

^3^ Laboratory of Bioinformatics, Wageningen UR, Wageningen, The Netherlands

* Corresponding author: [aaltjan.vandijk@wur.nl](mailto:aaltjan.vandijk@wur.nl)

**Supporting Information**

**SI Figure 1. Dependency of flowering time (vertical axis) on *FT* expression levels (horizontal axis) in various genetic backgrounds and various conditions, obtained in different studies**. Linear fits to the data are shown. Corresponding parameter values are presented in Table II and SI Table I. Table I indicates references for the different datasets. Note that results for one simultaneous fit of the various datasets shown in this figure are presented in SI Figure 8.

**SI Figure 2. Dependency of flowering time (vertical axis) on *FLC* expression levels (horizontal axis) in various genetic backgrounds and various conditions, obtained in different studies**. Linear fits to the data are shown. Corresponding parameter values are presented in Table II and SI Table I. Table I indicates references for the different datasets. Note that results for one simultaneous fit of the various datasets shown in this figure are presented in SI Figure 8.

**SI Figure 3. Dependency of flowering time (vertical axis) on *SVP* expression levels (horizontal axis) in various genetic backgrounds and various conditions, obtained in different studies**. Linear fits to the data are shown. Corresponding parameter values are presented in Table II and SI Table I. Table I indicates references for the different datasets. Note that results for one simultaneous fit of the various datasets shown in this figure are presented in SI Figure 8.

**SI Figure 4. Dependency of flowering time (vertical axis) on *LFY* expression levels (horizontal axis) in various genetic backgrounds and various conditions, obtained in different studies**. Linear fits to the data are shown. Corresponding parameter values are presented in Table II and SI Table I. Table I indicates references for the different datasets. Note that results for one simultaneous fit of the various datasets shown in this figure are presented in SI Figure 8.

**SI Figure 5. Dependency of flowering time (vertical axis) on *AGL24* expression levels (horizontal axis) in various genetic backgrounds and various conditions, obtained in different studies**. Linear fits to the data are shown. Corresponding parameter values are presented in Table II and SI Table I. Table I indicates references for the different datasets. Note that results for one simultaneous fit of the various datasets shown in this figure are presented in SI Figure 8.

**SI Figure 6. Histogram of Pearson R^2^ values for linear fits of data presented in Fig. 2 and SI Fig 1-5.**


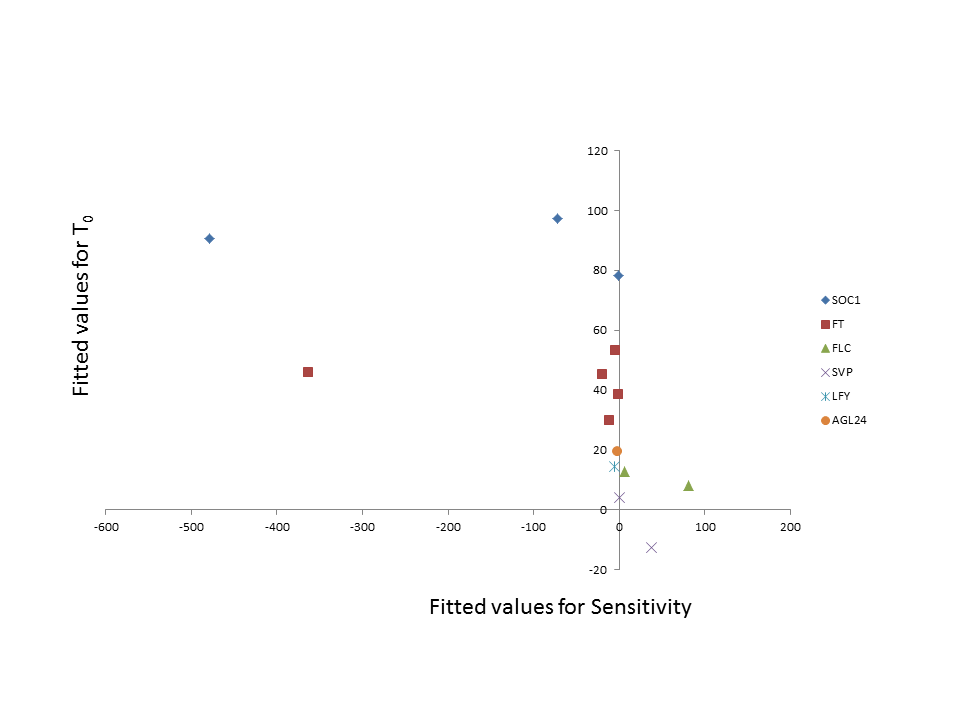


**SI Figure 7. Values obtained for parameters in linear fit.** Values of Sensitivity do not separate the different genes, whereas values of *T_0_* do. Hence, values of *T_0_* are rather similar to each other for a given gene.


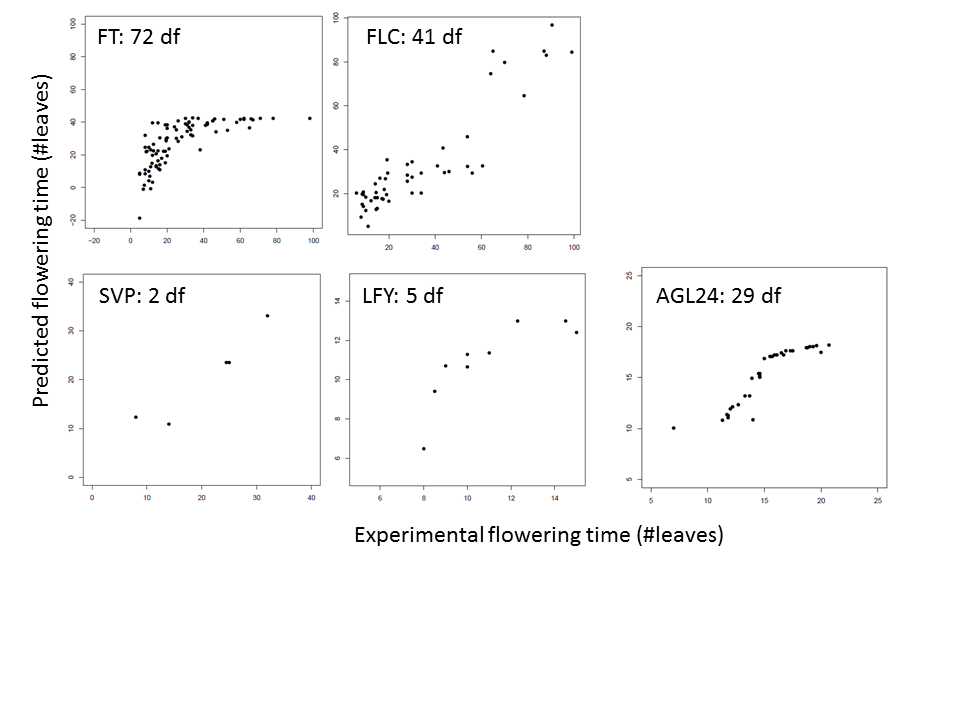


**SI Figure 8. Fitting combined datasets for each floral pathway integrator gene.** Plots show predicted vs. experimental flowering time using a single model for multiple expression datasets for the indicated genes. The various datasets were used in a combined fit with separate values of Sensitivity per dataset, but one overall *T_0_* per floral pathway integrator gene. Number of degrees of freedom in the resulting model is indicated.

**
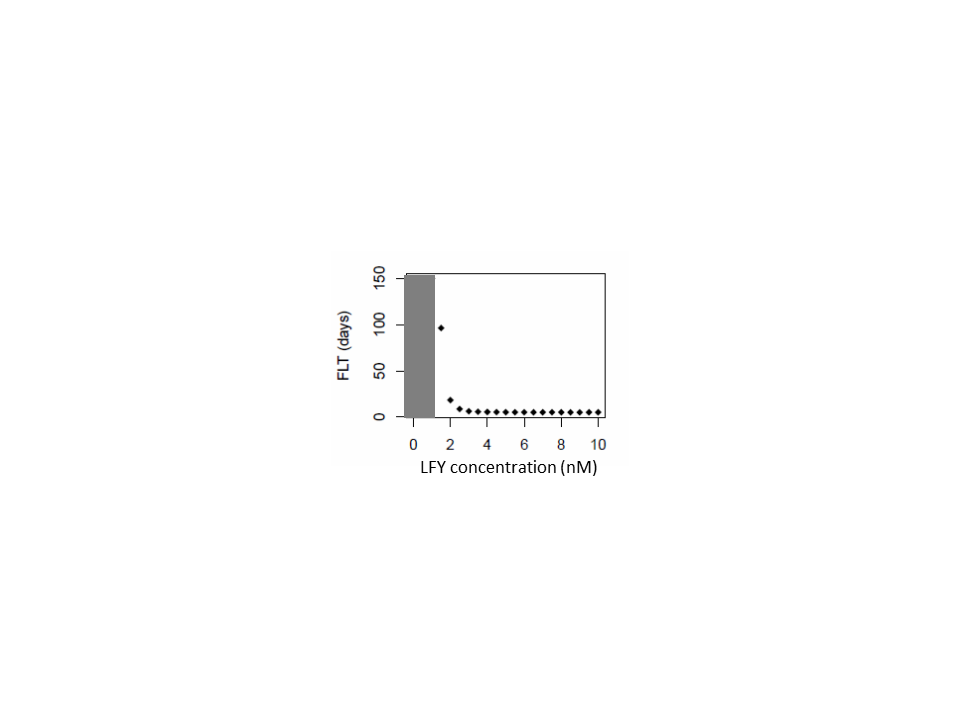
**

**SI Figure 9. Dependency of predicted flowering time (FLT) on *LFY* expression levels.** *LFY* expression was fixed at a given level and the resulting flowering time predicted by the ODE model [1] was recorded. For values of *LFY* below ~ 1nM, the model predicts that there is no flowering. Note that the values on the horizontal axis are not completely comparable with the values shown in Fig. 1, because in Figure 1 *LFY* concentration was not fixed, but was changed by modifying parameters in the equation describing *LFY* dynamics. The parameter modifications used in Fig. 1 did not result in an expression of *LFY* lower than ~1 nM in our model.

**SI Text: similarity of** *T_0_* **values**

As explained in the main text, values for *T_0_* obtained in different experiments for the same gene should be similar, even if a different normalisation has been used for the qPCR measurements. Visually the different *T_0_* values are indeed relatively similar (SI Fig. 7). Here we use a randomization procedure to assess whether this similarity is statistically significant. This analysis was performed by first calculating the coefficient of variation (standard deviation divided by absolute value of average) of the different *T_0_* values for each gene. This gives the following values: *AGL24*, 0.11; *SOC1*, 0.09; *LFY*, 0.19; *FT*, 0.37; *SVP*, 0.02; and *FLC*, 0.41. These values indicate that the standard deviation ranges from ~2% to ~40% of the average for the different genes. These values were then compared with values for the coefficient of variation obtained for randomized sets of *T_0_* values. These randomized sets were obtained by randomly redistributing the observed *T_0_* values over the different genes. After 1,000 randomizations, the number of cases in which for a given gene the same or lower value of coefficient of variation was obtained using these randomized sets, gives an estimate of the p-value for the similarity between the observed values of *T_0_*. These p-values are as follows: *AGL24*, 0.008; *SOC1*, 0.006; *FT*, 0.001; *LFY*, 0.05; *SVP*, 0.03; *FLC*, 0.01. These values indicate that indeed the observed similarities between values of *T_0_* from different datasets for the same gene are significant compared to random expectation.

For the sake of comparison, the same procedure was applied to values of *Sensitivity*. Here, as expected, the values of *Sensitivity* for a given gene were in general not similar to each other. P-values in this case were as follows: *AGL24*, 0.2; *SOC1*, 0.3; *FT*, 0.4; *LFY*, 0.01; *SVP*, 0.3; *FLC*, 0.02.

**SI Table I. Linear dependencies of flowering time on expression levels**

| **Gene** | **Normalization** | **Ref.** | **Sensitivity** | ***T_0_*** | **N** | **R^2^** | **p-value** | **Fig.** |
| --- | --- | --- | --- | --- | --- | --- | --- | --- |
| ***SOC1*** | scaled | [2] | -0.74 | 78.3 | 24 | 0.80 | 10^-9^ | 2A |
|  | actin | [3] | -72 | 97.5 | 6 | 0.76 | 0.02 | 2B |
|  | tubulin | [4] | -478.9 | 90.8 | 4 | 0.46 | 0.3 | 2C |
| ***FT*** | scaled | [5] | -0.39 | 46.3 | 4 | 0.999 | 10^-4^ | SI 1A |
|  |  | [5] | -0.25 | 31.7 | 4 | 0.46 | 0.3 | SI 1B |
|  |  | [6] | -0.27 | 37.4 | 3 | 0.99 | 0.06 | SI 1C |
|  | actin | [7-9] | -9.6 | 34.2 | 13 | 0.33 | 0.04 | SI 1D |
|  |  | [3] | -29.5 | 56.6 | 6 | 0.72 | 0.03 | SI 1E |
|  | tubulin | [10, 11] | -11.5 | 29.9 | 18 | 0.67 | 10^-5^ | SI 1F |
|  | IPP2 | [12] | -4.0 | 45.0 | 7 | 0.52 | 0.07 | SI 1G |
|  |  | [12] | -2.2 | 75.9 | 7 | 0.49 | 0.08 | SI 1H |
|  |  | [12] | -4.6 | 35.6 | 7 | 0.57 | 0.05 | SI 1I |
|  |  | [12] | -5.0 | 57.0 | 7 | 0.75 | 0.01 | SI 1J |
|  | UBQ10 | [13] | -56.0 | 62.6 | 4 | 0.93 | 0.03 | SI 1K |
|  |  | [14] | -1000 | 63 | 3 | 1.0 | 10^-16^ | SI 1L |
|  |  | [14] | -32.5 | 11.8 | 3 | 0.91 | 0.2 | SI 1M |
| ***FLC*** | scaled | [2] | 0.59 | 24.1 | 24 | 0.74 | 10^-8^ | SI 2A |
|  |  | [15] | 4.0 | 15.2 | 3 | 0.82 | 0.3 | SI 2B |
|  |  | [16] | 3.7 | 10.8 | 2 | NA | NA | SI 2C |
|  |  | [16] | 2.5 | 7.9 | 5 | 0.34 | 0.3 | SI 2D |
|  |  | [17] | 5.7 | 10.6 | 5 | 0.94 | 10^-3^ | SI 2E |
|  |  | [17] | 22.7 | 11.6 | 4 | 0.93 | 0.04 | SI 2F |
|  |  | [18] | 1.25 | 8.8 | 2 | NA | NA | SI 2G |
|  | actin | [8] | 81.0 | 8.1 | 5 | 0.85 | 0.03 | SI 2H |
| ***SVP*** | scaled | [6] | 0.29 | 4 | 2 | NA | NA | SI 3A |
|  | tubulin | [11] | 37.2 | -12.5 | 3 | 0.999 | 0.02 | SI 3B |
| ***LFY*** | scaled | [18] | -6.7 | 16.7 | 2 | NA | NA | SI 4A |
|  |  | [16] | -5.2 | 16.4 | 2 | NA | NA | SI 4B |
|  |  | [16] | -3.1 | 10.8 | 5 | 0.67 | 0.09 | SI 4C |
| ***AGL24*** | scaled | [19] | -4.2 | 20.6 | 10 | 0.96 | 10^-7^ | SI 5A |
|  |  | [19] | -0.74 | 16.6 | 20 | 0.96 | 10^-14^ | SI 5B |
|  |  | [6] | -0.12 | 21.5 | 3 | 0.80 | 0.3 | SI 5C |

**^a^** Values for parameters in linear fit T = Sensitivity*ExpressionLevel + *T_0_* for data shown in Fig. 2 and SI Figures 1-5. Normalization method used in the different datasets is indicated (scaled means normalization by scaling with wildtype and/or maximum expression value). N indicates number of datapoints in a given dataset. R^2^ indicates the Pearson R^2^ value for a linear fit of the data, and p-value the p-value of the linear model. NA indicates cases for which N=2 and hence R^2^ and p-value are not reported. Fig. indicates in which figure the dataset and fitted model is displayed. Each of the datasets for which results of are summarized in this table, is shown in supplementary datafile SIdataFile.xlsx.

**Additional datafiles:**

**Supplementary Datasheet 2:**  xlsx file containing the various literature-derived datasets analysed in this paper. Each dataset contains pairs of expression level – flowering time measured in various genetic backgrounds.

**Supplementary Datasheet 3:** example R-code used for analysis

**References**

1. Leal Valentim, F., et al., *A quantitative and dynamic model of the Arabidopsis flowering time gene regulatory network.* PLoS One, 2015. **10**(2): p. e0116973.

2. El-Din El-Assal, S., et al., *The role of cryptochrome 2 in flowering in Arabidopsis.* Plant Physiol, 2003. **133**(4): p. 1504-16.

3. Gunl, M., et al., *Analysis of a post-translational steroid induction system for GIGANTEA in Arabidopsis.* BMC Plant Biol, 2009. **9**: p. 141.

4. Liu, C., et al., *Direct interaction of AGL24 and SOC1 integrates flowering signals in Arabidopsis.* Development, 2008. **135**(8): p. 1481-91.

5. Mizoguchi, T., et al., *Distinct roles of GIGANTEA in promoting flowering and regulating circadian rhythms in Arabidopsis.* Plant Cell, 2005. **17**(8): p. 2255-70.

6. Li, D., et al., *A repressor complex governs the integration of flowering signals in Arabidopsis.* Dev Cell, 2008. **15**(1): p. 110-20.

7. Zuo, Z., et al., *Blue light-dependent interaction of CRY2 with SPA1 regulates COP1 activity and floral initiation in Arabidopsis.* Curr Biol, 2011. **21**(10): p. 841-7.

8. Yang, H., et al., *A companion cell-dominant and developmentally regulated H3K4 demethylase controls flowering time in Arabidopsis via the repression of FLC expression.* PLoS Genet, 2012. **8**(4): p. e1002664.

9. Fornara, F., et al., *Arabidopsis DOF transcription factors act redundantly to reduce CONSTANS expression and are essential for a photoperiodic flowering response.* Dev Cell, 2009. **17**(1): p. 75-86.

10. Endo, M., et al., *CRYPTOCHROME2 in vascular bundles regulates flowering in Arabidopsis.* Plant Cell, 2007. **19**(1): p. 84-93.

11. Nefissi, R., et al., *Double loss-of-function mutation in EARLY FLOWERING 3 and CRYPTOCHROME 2 genes delays flowering under continuous light but accelerates it under long days and short days: an important role for Arabidopsis CRY2 to accelerate flowering time in continuous light.* J Exp Bot, 2011. **62**(8): p. 2731-44.

12. Sawa, M. and S.A. Kay, *GIGANTEA directly activates Flowering Locus T in Arabidopsis thaliana.* Proc Natl Acad Sci U S A, 2011. **108**(28): p. 11698-703.

13. Tseng, T.S., et al., *SPINDLY and GIGANTEA interact and act in Arabidopsis thaliana pathways involved in light responses, flowering, and rhythms in cotyledon movements.* Plant Cell, 2004. **16**(6): p. 1550-63.

14. Wu, J.F., Y. Wang, and S.H. Wu, *Two new clock proteins, LWD1 and LWD2, regulate Arabidopsis photoperiodic flowering.* Plant Physiol, 2008. **148**(2): p. 948-59.

15. Niu, L., et al., *Regulation of flowering time by the protein arginine methyltransferase AtPRMT10.* EMBO Rep, 2007. **8**(12): p. 1190-5.

16. He, Y., et al., *Nitric oxide represses the Arabidopsis floral transition.* Science, 2004. **305**(5692): p. 1968-71.

17. Jiang, D., et al., *Arabidopsis relatives of the human lysine-specific Demethylase1 repress the expression of FWA and FLOWERING LOCUS C and thus promote the floral transition.* Plant Cell, 2007. **19**(10): p. 2975-87.

18. Wang, B., et al., *UGT87A2, an Arabidopsis glycosyltransferase, regulates flowering time via FLOWERING LOCUS C.* New Phytol, 2012. **194**(3): p. 666-75.

19. Yu, H., et al., *AGAMOUS-LIKE 24, a dosage-dependent mediator of the flowering signals.* Proc Natl Acad Sci U S A, 2002. **99**(25): p. 16336-41.
