# Supplementary material for: Floral pathway integrator gene expression mediates gradual transmission of environmental and endogenous cues to flowering time
Source: PeerJ. 2017 Apr 19;5:e3197. doi: 10.7717/peerj.3197 (PMC5399868; doi:10.7717/peerj.3197)
Supplement: Supplemental Information 2 [file peerj-05-3197-s003.docx]

Example R-script for analysis of SOC1 data:

#reading datafiles; these data are included in SI file SIdataFile.xls

dat1<-read.table("eldinelassal2003")

dat2<-read.table("gunl2009")

dat3<-read.table("liu2008agl24soc1")

#Fitting first dataset

layout(matrix(c(1,1,2,3), 2, 2, byrow = TRUE))

plot(dat1[,1],dat1[,2],xlim=c(0,100),ylim=c(0,100),xlab="ExpressionLevel",ylab="Flowering time (#leaves)")

r<-cor(dat1[,1],dat1[,2])

r**2

cor.test(dat1[,1],dat1[,2])

mod1=lm(dat1[,2]~dat1[,1])

abline(mod1$coef)

mtext(side=3,paste("y =",round(mod1$coef[2],2),"x +",round(mod1$coef[1],2)))

#Fitting second dataset

plot(dat2[,1],dat2[,2],xlim=c(0,1.5),ylim=c(0,100),xlab="ExpressionLevel",ylab="Flowering time (#leaves)")

r<-cor(dat2[,1],dat2[,2])

r**2

cor.test(dat2[,1],dat2[,2])

mod2=lm(dat2[,2]~dat2[,1])

mtext(side=3,paste("y =",round(mod2$coef[2],2),"x +",round(mod2$coef[1],2)))

abline(mod2$coef)

#Fitting third dataset

plot(dat3[,1],dat3[,2],xlim=c(0,0.1),ylim=c(0,100),xlab="ExpressionLevel",ylab="Flowering time (#leaves)")

r<-cor(dat3[,1],dat3[,2])

r**2

cor.test(dat3[,1],dat3[,2])

mod3=lm(dat3[,2]~dat3[,1])

mtext(side=3,paste("y =",round(mod3$coef[2],2),"x +",round(mod3$coef[1],2)))

abline(mod3$coef)

#Subsequently, fitting one combined model

y1<-dat1[,2]

x1<-dat1[,1]

y2<-dat2[,2]

x2<-dat2[,1]

y3<-dat3[,2]

x3<-dat3[,1]

y <- c(y1,y2,y3); x <- c(x1,x2,x3)

n1 <- length(x1)

n2 <- length(x2)

n3 <- length(x3)

lcon1 <- rep(c(1,0,0), c(n1,n2,n3))

lcon2 <- rep(c(0,1,0), c(n1,n2,n3))

lcon3 <- rep(c(0,0,1), c(n1,n2,n3))

#Combined fit with common 'b' parameter, other parameters separate

combi<-nls(y ~ a1*lcon1*x + a2*lcon2*x + a3*lcon3*x + b,

start = list(a1=1,a2=1,a3=1,b=0))

summary(combi)

predict(combi)

plot(y,predict(combi),pch=19,xlim=c(0,100),ylim=c(0,100))

cor.test(y,predict(combi))
